# Acute Q fever in an Omani presenting with a febrile movement disorder – A Case Report

**DOI:** 10.1016/j.idcr.2023.e01861

**Published:** 2023-07-25

**Authors:** Zoheb Jaffer Hussein, Abdul Salam AL Jalboubi, Ibrahim Al Busaidi

**Affiliations:** a Oman Medical Specialty Board, Internal Medicine Residency Program, Muscat, Oman; b Infectious Diseases Unit, Department of Medicine, Sultan Qaboos University Hospital, SQU, Muscat, Oman

**Keywords:** Q fever, Hemifacial spasm, *Coxiella burnetii*, Movement disorder mimic, Neurological manifestations of Q fever

## Abstract

Q fever is a zoonotic infection caused by the organism *Coxiella burnetii*. It is transmitted by contracting the organism from it is most known reservoirs which include cattle, sheep, and goats. It is an endemic disease with very few cases reported in the Arabian Peninsula. It can present with a wide range of clinical presentations; however, the neurological manifestation of Q fever is rare and overlooked hence we present a case report of a 38-year-old man who presented with fever, headache, and jerky movements. He had a significant exposure to livestock animals mainly goats and sheep. The work up was negative apart from positive *Coxiella burnetii* serology and serum PCR suggestive of acute Q fever. He had shown remarkable improvement after a course of antimicrobial therapy with complete resolution of fever and neurological symptoms. Our case report highlights the importance considering Q fever as a cause of unusual neurological symptoms in Q fever endemic areas and patients with a significant zoonotic exposure.

## Highlights

• Q fever-related neurological manifestations are rare and underreported.
• Movement disorder-like symptoms is a rare manifestation of Q fever.
• Q fever serology should be considered in cases of unusual neurological symptoms.
• Early diagnosis and therapy are paramount to prevent long term neurological sequelae.

## Introduction

Q fever is a ubiquitous zoonotic infection caused by *Coxiella burnetii*, an obligate intracellular gram-negative bacterium. Cattle, sheep, and goats are among the main recognized reservoirs of *C. burnetii* [1], [2]. Human infection is believed to occur by the inhalation of aerosols contaminated with *C. burnetii*. Other reported modes of disease transmission namely ingestion of raw milk, infected tick bite, and person-to-person transmission [1], [3].

Clinical signs appear 1–3 weeks following infection, and early symptoms include flu-like illness, muscle pains, nausea, vomiting, diarrhea, night sweats, and abdominal or chest pains. The severity of Q fever ranges from asymptomatic to non-specific febrile syndrome or flu-like illness, pneumonia, hepatitis, endocarditis, vascular infection, osteoarticular infection, secondary hemophagocytic lymph histiocytosis (HLH) syndrome, thrombosis, lymphadenitis mimicking lymphoma, optic neuritis, polyradiculoneuritis, SIADH, mesangioproliferative glomerulonephritis, orchitis, acalculous cholecystitis, epididymitis and lastly on the extreme end of the spectrum multiorgan failure and death[6], [7], [8], [9], [10]. However, neurological involvement is reported to be rare, and it represents only (10%) of disease which includes severe headache (41%), confusion, focal neurological deficit, and rarely aseptic meningitis/meningoencephalitis (0.8%) [4], [5].

Here we present a case of acute Q fever with unusual neurological manifestations. In this case report we highlight one of the rare neurological manifestations of acute Q fever, clinical approach, and management.

## Case presentation

A 38-year-old Omani man, presented to the emergency department at Sultan Qaboos University Hospital (Oman) with a 9-day history of fever and bilateral electrical headache associated with fatigue, lightheadedness, slurring of speech and involuntary facial twitching, eye blinking, and upper limb jerky movement. The patient denied photophobia, phonophobia, nausea or vomiting, seizures, or behavioral changes. The patient reported a significant contact with farm animals including sheep, goats and cattle and reported consumption of unpasteurized milk.

In the emergency department, he was alert and oriented but noted to have episodic involuntary facial twitch bilaterally. He was febrile (39.6 °C) and tachycardic (107 beats per min). Blood pressure 133/96 mmHg, Respiratory rate was 18 breaths/min, and oxygen saturation was 99% on room air. A neurological exam revealed an equally reactive pupil, normal tone, normal deep tendon reflexes, and power of 5/5 in both upper and lower limbs. There were no signs of meningitis and rest of physical examination was otherwise normal.

The patient was empirically started on ceftriaxone and intravenous acyclovir to cover the possibility of acute bacterial meningitis or viral encephalitis.

Routine investigations showed raised C-reactive protein and mildly raised liver enzymes. Chest X-ray and brain MRI were normal. Electroencephalography showed no epileptic discharge. CSF analysis and microscopy were normal. CSF culture and viral screen were negative. An extended panel for common infectious diseases was negative (Table 1). Q fever serology came back positive for IgM phase II and negative for IgG phase II and IgM phase I antibodies. *Coxiella burnetii* PCR was positive in serum. Patient was started on oral doxycycline 100 mg twice per day for 14 days. On follow up patient’s symptoms resolved and repeated Q fever serology was positive for IgG and IgM phase II antibodies representing seroconversion and acute Q fever infection.

**Table 1:** Laboratory Investigations at the time of admission and after 3 weeks. Key – ALT (Alanine aminotransferase), AST (Aspartate aminotransferase), CSF (Cerebrospinal fluid), CRP (C-reactive protein), CMV (Cytomegalovirus), EBV (Epstein-Barr Virus).

Test	At admission	After 3 weeks	Reference Range
Hemoglobin	13.2	14.5	11–14.5 g/dL
White Cell Count	2.6	3.7	2.4–9.5 × 10^9^/L
Neutrophils	1.6	1.5	1.0–4.8 × 10^9^/L
Platelets	149	152	150–450 10^9/L
CRP	105	< 1	0–5 mg/L
ALT	**61**	**42**	**0–40 U/L**
AST	**92**	**29**	**0–41 U/L**
HIV 1&2 (Abs/Antigen)	negative	-	-
CMV Serology	IgG positive, IgM negative	-	-
Dengue	IgG, IgM, and NS1 negative	**-**	**-**
Brucella	Negative (melitensis and abortus)	-	Negative - Titre < 1:20
EBV	VCA-IgG +ve, VCA-IgM negative	-	-
CSF viral screen	HSV, enterovirus, VZV, Mumps and parechovirus PCR negative	-	-
CSF culture	Negative	-	
CSF microscopy	Leukocyte 1, Erythrocyte 0	-	Leukocyte 0–10/cu mm
*Coxiella burnetii* Serology	**IgM phase II positive**	**IgG and IgM phase II positive**	**-**
*Coxiella burnetii* PCR (serum)	**Detected**		**-**

## Discussion

There are some similarities in our patient’s neurological symptoms with what has been previously reported in case reports of Q fever with neurological manifestation. However, data about movement disorder like presentation in Q fever is scarce. Our patient presented with headache, speech disturbance, hemifacial spasm, and features suggestive of a movement disorder with normal brain imaging, CSF studies and EEG. He had a complete resolution of symptoms after a course of doxycycline. Patient had a positive Q fever with evidence of acute seroconversion and positive *Coxiella burnetii* PCR in the serum confirming the diagnosis of acute Q fever as the cause of his neurological symptoms.

The difference in our case report is that Q fever is usually present in areas where the infection is endemic, which is not the case in the Arabic peninsula [11], [12], [13]. Our case reveals a rare manifestation of uncommon infection in our region which commonly presents as fever of unknown origin, hepatitis, culture-negative endocarditis, or neurological sequelae secondary to septic emboli [11], [12], [13], [14], [15]. Q fever presenting with neurological manifestation is uncommon. Q fever has been reported to cause various of neurological manifestation broadly specified to meningitis, encephalitis, encephalomyelitis, Gullian-Barre Syndrome, toxic confusional state, behavioral changes, cranial nerve involvement, and mononeuritis multiplex [4], [12], [13], [14], [15]. The possible pathogenesis of neurological manifestations *Coxiella burnetii* is related to direct CNS tissue damage, septic emboli, or possible circulating immune complexes [14], [15].

Q fever-related neurological manifestations are not uncommon, however, due to a lack of awareness and low index of suspicion it remains a rare and underreported entity. If not treated in a timely fashion, 10–20% of cases may have neurological sequelae and 1–2% may have chronic indolent Q fever [15]. Due to high pathogenicity and tendency to progress, it is paramount to have a high index of suspicion to make early diagnosis and effective antimicrobial therapy [15]. Based on the findings from our case, testing for Q fever is recommended for unusual neurological symptoms in patients from endemic regions or those with a significant zoonotic exposure.

## Conclusion

Our case report highlights that Q fever should be considered as a cause of acute neurological disease, and serological testing should be performed in all cases of meningoencephalitis, meningitis, peripheral neuropathy, or any unusual neurological symptoms in patients from endemic places or those with risk factors. Despite rarity of neurological manifestations, a high index of suspicion and early treatment is a key in preventing long term neurological sequelae. Further studies are required to better understand this rare entity of Q fever and its pathogenicity.

## Consent

Consent for publication of the case was granted by the patient.

## Funding

This manuscript has no funding source outside the authors.

## CRediT authorship contribution statement

Dr. Zoheb Jaffer Hussein and Dr. Abdul Salam AL Jalboubi wrote the case reports and major parts of the manuscript. Dr. Al-Busaidi reviewed and revised the manuscript and contributed to discussion part.

## Declaration of Competing Interest

The authors have no conflicts of interest to declare.
